# Extreme stratospheric wave activity as harbingers of cold events over North America

**DOI:** 10.1038/s43247-023-00845-y

**Published:** 2023-05-27

**Authors:** Xiuyuan Ding, Gang Chen, Pengfei Zhang, Daniela I. V. Domeisen, Clara Orbe

**Affiliations:** 1 Department of Atmospheric and Oceanic Sciences, University of California, Los Angeles, Los Angeles, CA USA; 2 Department of Meteorology and Atmospheric Science, The Pennsylvania State University, University Park, PA USA; 3 University of Lausanne, Lausanne, Switzerland; 4 Institute for Atmospheric and Climate Science, ETH Zurich, Zurich, Switzerland; 5 NASA Goddard Institute for Space Studies, New York, NY USA

**Keywords:** Atmospheric dynamics, Natural hazards

## Abstract

Extreme cold events over North America such as the February 2021 cold wave have been suggested to be linked to stratospheric polar vortex stretching. However, it is not resolved how robustly and on which timescales the stratosphere contributes to the surface anomalies. Here we introduce a simple measure of stratospheric wave activity for reanalyses and model outputs. In contrast to the well-known surface influences of sudden stratospheric warmings (SSWs) that increase the intraseasonal persistence of weather regimes, we show that extreme stratospheric wave events are accompanied by intraseasonal fluctuations between warm and cold spells over North America in observations and climate models. Particularly, strong stratospheric wave events are followed by an increased risk of cold extremes over North America 5–25 days later. Idealized simulations in an atmospheric model with a well-resolved stratosphere corroborate that strong stratospheric wave activity precedes North American cold spells through vertical wave coupling. These findings potentially benefit the predictability of high-impact winter cold extremes over North America.

## Abstract (web summary)

Extreme stratospheric wave events are associated with intraseasonal fluctuations between cold and warm spells over North America, according to an analysis of observations and climate model simulations.

## Introduction

As global warming is expected to reduce the frequency of severe cold spells, the causes of recent winter cold extremes in the Northern Hemisphere have attracted much public attention and scientific debate^1–6^. Notably, the exceptional North American cold wave in February 2021 was suggested to be related to the sudden stratospheric warming (SSW) in January 2021^7,8^, which was further proposed to be linked to Arctic sea ice loss under global warming^3,4^. However, numerical weather forecasts for the February 2021 cold wave suggest that the January 2021 SSW event exerted only a limited influence on surface temperature^5,9,10^. Given the disproportionate impacts of winter cold extremes on energy and society^11^, an improved understanding of the role of the stratosphere in the predictability of surface cold spells is warranted.

There exist different types of stratospheric impacts on surface air temperature (SAT). SSWs feature an abrupt deceleration and reversal of the zonal wind in the winter stratosphere primarily due to planetary wave breaking and wave absorption, which is followed by downward propagation of negative zonal wind anomalies to the lower stratosphere on weekly to monthly timescales^12–20^. The surface composite of SSWs is characterized by an anomalous high pressure center near Greenland, with anomalous cooling over northern Eurasia and warming over eastern Canada that resemble the negative phase of the Arctic Oscillation (AO). In contrast, extreme stratospheric wave events, featuring negative meridional eddy heat flux due to planetary wave reflection, are instantaneously linked to the positive phase of the North Atlantic Oscillation (NAO) and anomalous North American cooling^21–23^.

These two types of stratospheric impacts on the surface can be distinguished by the sign of lower-stratospheric meridional heat flux during the recovery stage of SSWs^24,25^, clustering analysis^3,26–28^, or empirical orthogonal function (EOF) analysis^29,30^. Particularly, Cohen et al.^3^ argues that stratospheric polar vortex stretching involving planetary wave reflection is linked to North American cold spells such as the February 2021 Texas cold wave. However, the Alaskan ridge weather regime associated with widespread severe North American cold does not show a dependency on stratospheric vortex strength^27^. Stratospheric wave reflection events are further associated with an evolution from a Pacific trough regime with surface warm anomalies over North America to an Alaskan ridge regime that favors North American cold^31^. In contrast to circulation regimes, the relevant stratosphere-troposphere coupling can be characterized as an intraseasonal variability mode^30^. As the wave reflection events in these studies are often based on the lower-stratospheric (i.e., 100 hPa) circulation patterns that may be strongly related to tropospheric variability, the contribution of stratospheric variability to North American cold snaps remains unclear.

Identifying the surface signals of stratospheric variability is also hindered by large internal variability in both the stratosphere and troposphere. Distinct surface signals are found to follow different types of SSW events, such as the displacement of a polar vortex off the pole versus the split of a polar vortex into two smaller vortices^32,33^. Strong tropospheric weather variability can obscure the predictability from the downward propagation of signals, with only about two-thirds of SSWs being followed by visible downward influences featured by negative AO^34–36^. Using large ensembles from a climate model that differ only by small changes to the initial conditions, previous studies have found that even a 100-member ensemble may be insufficient to detect the surface influences of stratospheric variability under climate change, due to the large internal atmospheric variability^37,38^. A previous study finds that the February 2021 North American cold event was largely affected by unpredictable internal atmospheric variability^5^. We will thus use both observations and climate model ensembles to evaluate the robustness and mechanisms of the contributions of extreme stratospheric events to surface temperature anomalies.

In this study, we will first present the surface fingerprints of extreme stratospheric wave activity in observations and the historical simulations from 30 climate models from the Coupled Model Intercomparison Project Phase 6 (CMIP6), showing that strong stratospheric wave events are consistently followed by North American cold anomalies 5–25 days later. This indicates that strong stratospheric wave activity can serve as a sub-seasonal predictor for cold air outbreaks over North America. In contrast to the persistent weather regimes associated with stratospheric polar vortex events^12–19^ or lower-stratospheric wave reflection events^3,26^, we substantiate an emerging linkage between extreme stratospheric wave events and the intraseasonal fluctuations between warm and cold snaps over North America^29–31^. We further support this linkage through idealized nudging simulations in the Specified Chemistry Whole Atmosphere Community Climate Model (SC-WACCM4)^39^, which suggests that the vertical wave coupling plays a key role in the North American cold extremes following strong stratospheric wave events.

## Results

### Surface signatures of extreme stratospheric wave events

**Fig. 1:**
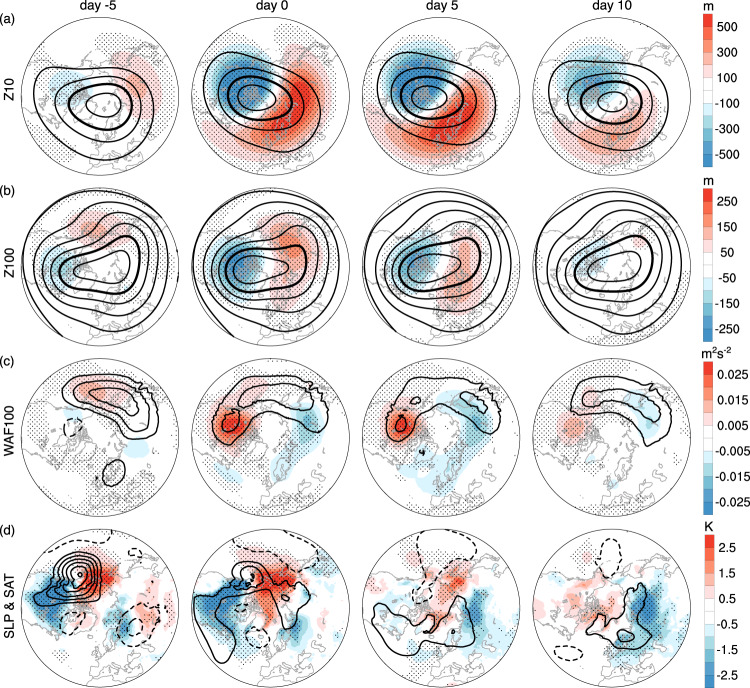
Weak stratospheric wave events in ERA5 reanalysis. Composites of days −5, 0, 5, and 10 with respect to the onset of weak stratospheric wave events: **a** 10 hPa geopotential height (contours at 500 m intervals, 29000 m contour bolded, anomalies shaded). **b** 100 hPa geopotential height (contours at 200 m intervals, 15200 m contour bolded, anomalies shaded). **c** 100 hPa vertical component of Plumb wave activity flux (contours at 0.01 m^2^s^−2^ intervals, anomalies shaded). **d** anomalous SLP (contours at 2 hPa intervals) and SAT (shading). The time evolution is smoothed by a 5-day running average (i.e., day −5 is the average of days −7 to −3). The weak wave events are defined by the 5th percentile of the first principal component of the zonally asymmetric component of 10 hPa geopotential height. See details in Methods. Stippling indicates the regions where the anomalies are significant at the 95% confidence level based on the Student’s *t*-test.

We begin by characterizing the surface signatures of extreme stratospheric wave events. Strong and weak wave events are identified by extreme percentiles of the wave index, which is defined as the leading principal component of the zonally asymmetric component of geopotential height at 10 hPa for the extended boreal winter from November to March in ERA5 reanalysis and CMIP6 models (see details in Methods). The corresponding EOF mode of 10-hPa geopotential height features a transient planetary wave-1 pattern, with the positive phase amplifying the climatological wave pattern through constructive wave interference and the negative phase weakening the climatological wave via destructive interference^29^ (Supplementary Fig. 1). Consecutive days above the 95th percentile of the wave index are referred to as strong wave events, and the days below the 5th percentile are termed weak wave events. No minimum duration is required for an event. We note that the EOF pattern of transient waves does not fully align with the climatological wave pattern, and a weak wave event may still produce a polar vortex stretching (Fig. 1a). Compared with previous definitions of stratospheric wave events such as eddy heat fluxes^23^, cluster analysis^3,26,28^, or temporal filtering^30^, we believe that the simplicity of this stratospheric wave event definition is appealing, especially in regard to model intercomparison and evaluation. The consistency among different data sets provides strong support for surface signatures of extreme stratospheric wave events.

The weak wave event composite displays a stretching of the stratospheric polar vortex at 10 hPa towards North America from day −5 to day 0, with an anomalous ridge over Eurasia and a trough over North America (Fig. 1a), resembling the 10-hPa composite of the tropospheric Alaskan ridge weather regime^27^. The polar vortex in the lower stratosphere (100 hPa) is also stretched, but with an anomalous ridge over Alaska and Eastern Siberia and a trough over North America (Fig. 1b), similar to the circulation pattern observed for polar vortex stretching^3,26^. Interestingly, while the anomalous upward Plumb wave activity flux over North America and downward flux over Siberia are consistent with the vortex stretching (Fig. 1c), these anomalous fluxes differ from the vertical wave activity fluxes during polar vortex stretching disruptions^3,26^. Moreover, the anomalous cooling over North America and warming over Alaska and Eastern Siberia occur before and near the event onset (i.e., days −5 and 0) (Fig. 1d). Thus, the surface cooling may contribute to the weak stratospheric wave event rather than being an effect of the stratospheric event^40,41^. It is also noteworthy that the cold anomalies over northern Eurasia become more extensive from day −5 to day 10.

**Fig. 2:**
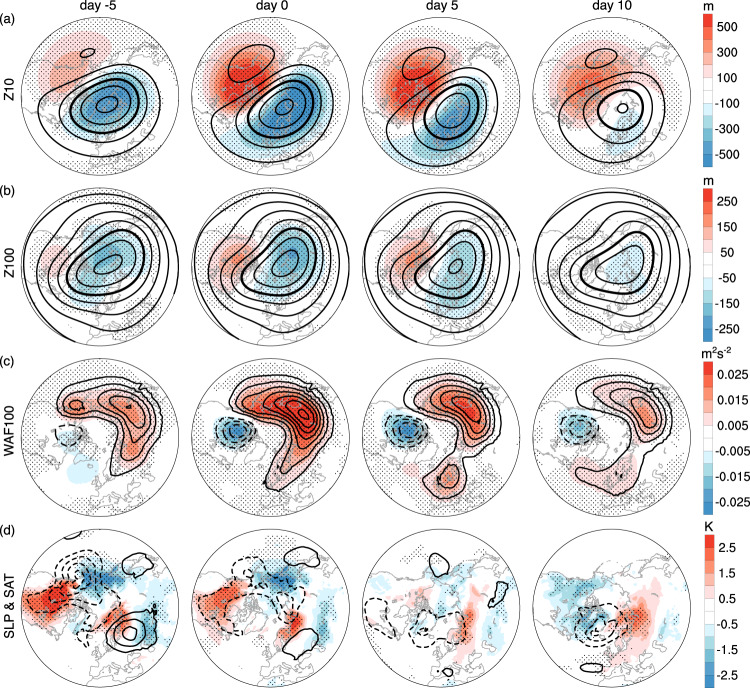
Strong stratospheric wave events in ERA5 reanalysis. As in Fig. 1, but for composites of strong stratospheric wave events. The strong wave events are defined by the 95th percentile of the first principal component of the zonally asymmetric component of 10 hPa geopotential height. See details in Methods.

The strong wave composite, in contrast, exhibits a displacement of the stratospheric polar vortex towards Eurasia at both 10 hPa and 100 hPa (Fig. 2a, b). The overall circulation anomalies of strong wave events are opposite to those of the weak wave events (Fig. 1a, b). Importantly, both the total and anomalous vertical wave activity fluxes are negative over northern North America and persist from the onset day to 10 days later, indicating a local feature of planetary wave reflection (Fig. 2c). This feature coincides with a transition from anomalous warming over North America before the event onset to cooling 10 days after the onset, as well as the development of a cyclonic anomaly in sea level pressure (SLP) over Greenland 5 to 10 days after the event onset (Fig. 2d). We also observe that warm anomalies over northern Eurasia are amplified and persist from day −5 to day 10. The relatively long timescale of Eurasian warm anomalies indicates that they could be remnants of positive AO, which is a surface response to the anomalously strong polar vortex prior to strong wave events (Supplementary Fig. 2). On the other hand, weak wave events exhibit much smaller zonal wind anomalies than strong wave events, implying an asymmetry between weak and strong stratospheric wave events.

As the number of extreme stratospheric wave events are limited in observations (1.25 weak and 1.31 strong events per year on average for 1950–2021; see the statistics in Methods), we have also analyzed the historical simulations in 30 CMIP6 models (Supplementary Figs. 3, 4). The general characteristics of the extreme wave events in CMIP6 models are strikingly similar to reanalysis in Figs. 1, 2. These reanalysis and model results consistently indicate that weak stratospheric wave events are associated with North American cooling before and near the polar vortex stretching, and that strong stratospheric wave activity is characterized by a vortex displacement to Eurasia and downward wave activity fluxes over northern North America, followed by North American cooling about 10 days later.

### Strong wave events increase the risk of North American cold extremes

**Fig. 3:**
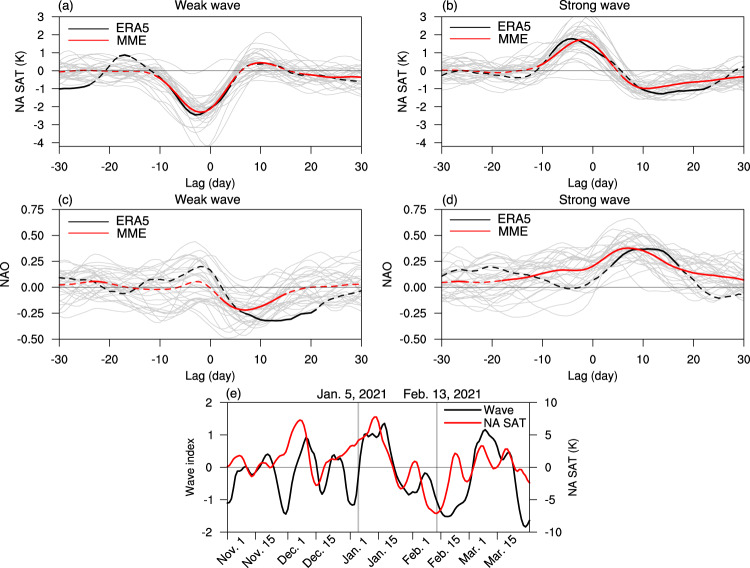
Evolution of North American SAT anomalies linked to extreme stratospheric wave events. **a**, **b** Composites of North American SAT (NA SAT) anomalies for weak (**a**) and strong (**b**) stratospheric wave events in ERA5 reanalysis and CMIP6 models. ERA5 is depicted as black lines, the CMIP6 multi-model ensemble (MME) means in red, and individual models in light gray. **c**, **d** As in (**a**, **b**), but for the NAO index. **e** Evolution of the stratospheric wave index and NA SAT anomalies for winter 2020/2021. Solid parts of the lines for ERA5 and CMIP6 MME in (**a**–**d**) represent the composites significant at the 95% confidence level based on the Student’s *t*-test. Gray lines in (**e**) denote the onset date of the SSW on January 5, 2021, and the lowest NA SAT on February 13, 2021, for the winter. NA SAT anomalies are averaged over the land regions of 40^∘^–70^∘^N, 70^∘^–130^∘^W. The NAO index is defined as the SLP difference between 20^∘^–55^∘^N, 90^∘^W–60^∘^E and 55^∘^–90^∘^N, 90^∘^W–60^∘^E.

We next compare the evolution of SAT anomalies over North America for weak and strong stratospheric wave events in ERA5 and CMIP6 models (Fig. 3a, b), confirming the transitions between warm and cold spells over North America (Figs. 1d and 2d). The cold SAT anomalies following strong wave events are also consistent with the positive NAO anomalies from day 0 to day 15 (Fig. 3d), which correspond to the cyclonic anomaly in SLP near Greenland and associated cold air advection over North America (Fig. 2d). Since the surface cold anomalies over North America take place 5–25 days after the stratospheric event onset, the strong stratospheric wave events can be used as a sub-seasonal predictor for North American cold extremes. It is then tempting to ask whether extreme stratospheric wave events may be related to the February 2021 Texas cold wave, given its proposed linkage to a stratospheric polar vortex stretching^3^. The daily evolutions of the stratospheric wave index and the SAT anomaly for winter 2020/2021 are plotted in Fig. 3e. Following the strong wave activity around January 18, 2021, cold SAT anomalies are observed from late January to mid-February concurrently with weak stratospheric wave activity. While the linkage between individual cold events and stratospheric wave activity should be interpreted with caution, these are largely consistent with the composite analysis in Fig. 3a, b. But given that the cold anomalies in response to stratospheric wave events are mostly north of 40^∘^N (Fig. 2d), the strong stratospheric wave activity around January 18, 2021, is unlikely a primary contributor to the deep cold in 2021 that reached Texas (30^∘^N), consistent with previous studies^5,9^.

**Fig. 4:**
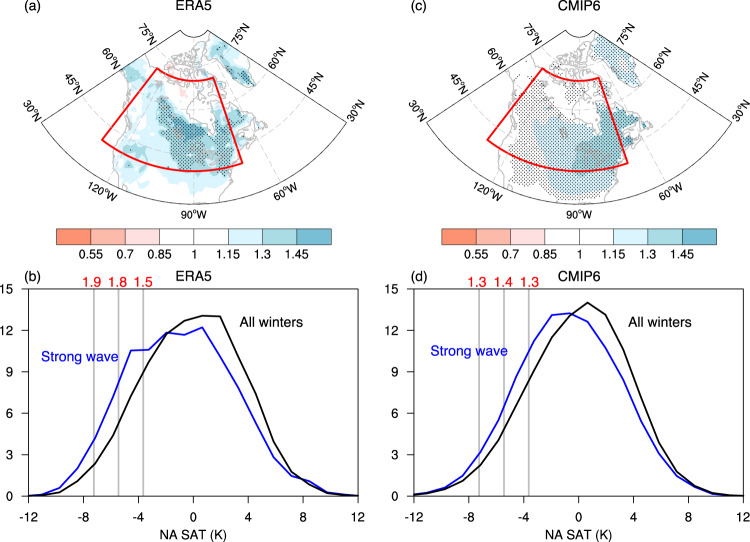
Risk ratio of extreme cold days and probability density function (PDF) of NA SAT anomalies following strong stratospheric wave events. **a**, **b** The spatial pattern of the risk ratio of extreme cold days (**a**) and the PDF of NA SAT anomalies (**b**) in ERA5 during days 5–25 after the onset of strong stratospheric wave events, compared with the statistics of all winters. The risk ratio in (**a**) is defined as the probability of cold days (i.e., SAT is at least 1.5 SD below its climatology) in days 5–25 divided by the probability of cold days in any random 21-day period in winter. Stippling indicates where the risk ratio is significant at the 95% confidence interval based on a Student’s *t*-test. **c**, **d** As in (**a**, **b**), but for CMIP6 models. The red boxes in (**a**) and (**c**) indicate the region where the NA SAT anomalies are calculated. The vertical gray lines in (**b**) and (**d**) denote −1, −1.5, and −2 SD of NA SAT anomalies in all the winter days, and the values in red depict the risk ratios of the exceedance frequency due to strong wave events.

We will hereafter focus on strong stratospheric wave events, as they are potentially useful for extended-range forecasting of cold events, and their impacts on winter cold extremes are quantified by the risk ratio of extreme cold days. An extreme cold day is defined as a day when the local SAT lies at least 1.5 standard deviations (SD) below its climatology. Fig. 4a shows the risk ratio of extreme cold days for 5–25 days after strong stratospheric wave events. Compared to all the winter days, strong stratospheric wave events enhance the risk of extreme cold days by about 30% across much of Canada and the Northeast U.S. Moreover, the probability density function (PDF) for the area-averaged SAT anomalies over North America during 5–25 days after strong wave events indicates a general shift towards colder SAT (blue) as compared to the PDF for all the winter days (black) (Fig. 4b). The risk ratios in the ERA5 reanalysis for the exceedance frequency below −1, −1.5, and −2 SD of the PDF are 1.5, 1.8, and 1.9, respectively. These observed characteristics are remarkably similar to the CMIP6 multi-model means, albeit with lower risk ratios in CMIP6 (Fig. 4c, d). Furthermore, we examine the PDF of North American SAT for finer time windows (i.e., days 5–9, days 10–14, days 15–19, and days 20–24) in CMIP6. This confirms similar shifts towards colder SAT over shorter periods, with the largest cold anomalies for days 10–14 (Supplementary Fig. 5). These results provide consistent observational and modeling evidence that strong stratospheric wave events can increase the frequency of cold snaps over North America.

### Strong stratospheric wave events impact the surface via vertical wave coupling

**Fig. 5:**
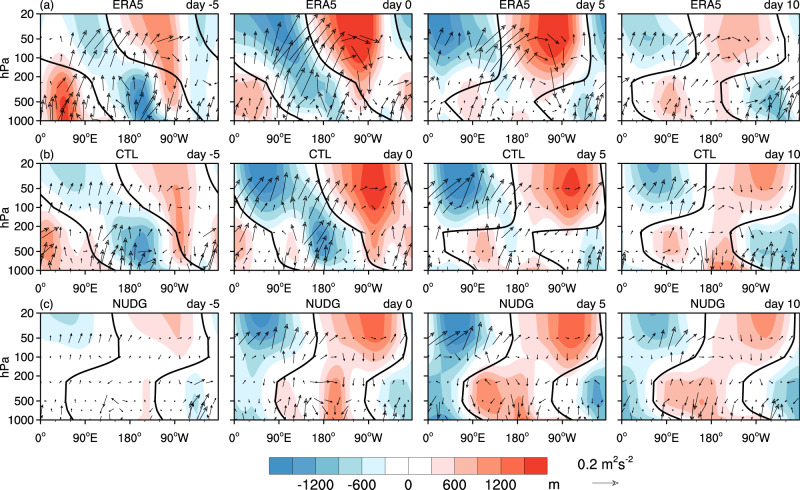
Vertical wave coupling during strong stratospheric wave events. **a** Composites of the zonally asymmetric component of anomalous geopotential height (shading) and the vertical and zonal components of anomalous Plumb wave activity flux (vector) averaged over 50^∘^–70^∘^N as a function of longitude and pressure on days −5, 0, 5 and 10 in ERA5. **b**, **c** As in (**a**), but for the CTL (**b**) and NUDG (**c**) experiments of SC-WACCM4. Black lines are zero contours of the wave-1 component of anomalous geopotential height, indicating the phase tilt of wave-1. To account for the smaller air density with decreasing pressure, the magnitude of the Plumb flux is scaled by (1000/*p*)^1/2^, and geopotential height is scaled by (*p*/1000)^1/2^, where *p* is pressure. The vertical component of the Plumb flux is also scaled by a factor of 200. See Supplementary Fig. 8 for the total field of anomalous height and absolute Plumb flux.

How does strong stratospheric wave activity influence the surface temperature? We first investigate the mechanisms in ERA5, using Plumb wave activity fluxes (See details in Methods) averaged over 50^∘^–70^∘^N as a function of longitude and pressure for days −5, 0, 5, and 10 (Fig. 5a). Over Siberia (roughly 90^∘^–135^∘^E), Plumb fluxes in the lower stratosphere are predominantly upward and eastward throughout the strong wave events, contributing to the amplification and persistence of an anomalous stratospheric ridge over North America from 5 days before the event onset to 5 days after onset. As the anomalous ridge intensifies, the initial westward tilt of the ridge with increasing altitude on day −5 transitions to a nearly barotropic structure starting at the event onset. After the event onset, the eastern edge of the anomalous ridge (or the trough from 90^∘^W to 0^∘^) displays an eastward tilt with increasing altitude, marking the region of downward wave activity flux and a tropospheric trough that develops over eastern North America from day 0 to day 10. This change in vertical phase line is also pronounced for the planetary wave-1 alone (black lines in Fig. 5). The amplification and increasingly more barotropic structure of the stratospheric ridge over North America from day −5 to day 5 might be thought of as local planetary wave reflection, although the zonal mean of vertical wave flux at 100 hPa is positive throughout the wave event (Fig. 2) and thus does not meet the criterion of planetary wave reflection for a given zonal mean background flow^42^.

To understand the contributions of stratosphere-troposphere coupling to the anomalous SAT, we further analyze two idealized simulations of an atmospheric global circulation model with a well-resolved stratosphere (i.e., control (CTL) and nudged (NUDG) runs in SC-WACCM4; See details in Methods). The NUDG run is the same as CTL, except that the model’s prognostic variables in the stratosphere are nudged to the corresponding stratospheric evolution in CTL while the underlying troposphere is allowed to evolve freely, with the nudging strength gradually decreasing to zero below 90 hPa. The evolution of the stratosphere in the NUDG run is almost the same as CTL, and thus the same stratospheric wave events are selected based on the 10 hPa wave index as in CTL (1.14 weak and 1.31 strong wave events per year on average).

**Fig. 6:**
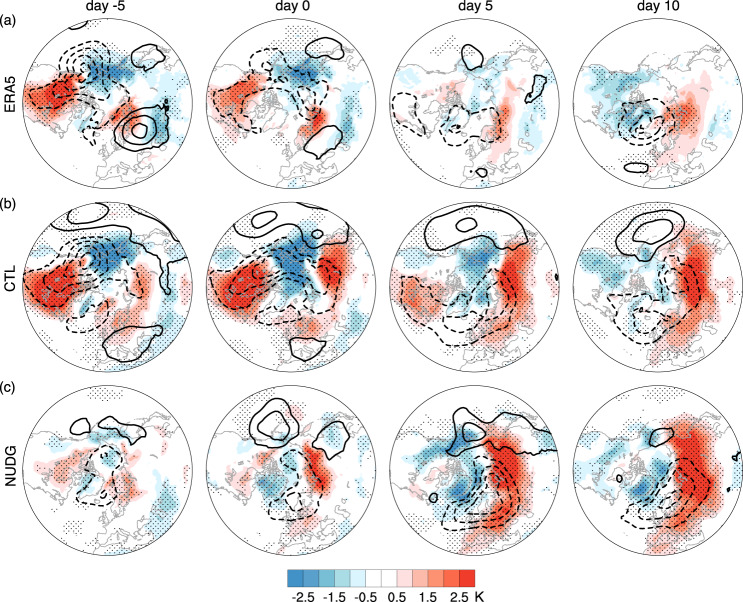
Surface signatures of strong stratospheric wave events in SC-WACCM4. Composites of anomalous SLP (contours at 2 hPa intervals) and SAT (shading) for the CTL (**b**) and NUDG (**c**) experiments of SC-WACCM4, as compared with ERA5 (**a**, same as Fig. 2d). Stippling indicates the regions where the SAT anomalies are significant at the 95% confidence level based on the Student’s *t*-test.

In the CTL experiment, the surface composite of strong wave events (Fig. 6b) is similar to that in ERA5 (Fig. 6a), with a transition from anomalous North American warming before the event onset to cooling by day 10, as well as the cyclonic anomaly in SLP near Greenland 5–10 days after the onset. While the stratospheric circulation in the NUDG simulation is almost the same as that in the CTL run, the small differences from CTL produce distinct trajectories in tropospheric weather due to the chaotic nature of weather, and thus the stratospheric wave events produced by nudging are decoupled from tropospheric weather precursors. Indeed, the NUDG experiment cannot reproduce the SAT and SLP anomalies before the event onset in CTL and reanalysis (Fig. 6). In contrast, as the stratospheric wave events often bear a large vertical scale, nudging the stratospheric component of a deep wave structure may produce the surface signatures of stratospheric waves due to vertical wave coupling. This is manifested by the North American cooling and Eurasian warming 10 days after the event onset, similar to CTL and reanalysis (Fig. 6).

We now link the distinct surface evolution in CTL and NUDG runs to vertical wave coupling. In the CTL run, an anomalous ridge over North America extends from the troposphere to the stratosphere and becomes increasingly barotropic from day -5 to day 5, and an anomalous tropospheric trough starts to emerge over Eastern North America 5 days after the event onset (Fig. 5b), similar to ERA5 (Fig. 5a). The NUDG experiment displays the same stratospheric evolution throughout the events as the CTL simulation by design, but the tropospheric precursors are absent on day −5, as tropospheric weather precursors are decoupled from the stratospheric evolution produced by nudging due to the chaotic behavior inherent in weather. Interestingly, the NUDG run exhibits a coherent eastward tilt of the ridge and trough anomalies with increasing height starting from the event onset, and therefore these tropospheric anomalies occurring after the event onset are plausibly linked to the stratospheric conditions nudged to the CTL simulation. This eastward tilt in wave phase after the event onset indicates that an anomalous tropospheric trough over Eastern North America below the anomalous stratospheric ridge (Fig. 5b, c), which, in turn, corresponds to the cyclonic anomaly in SLP over Greenland and cold air advection over North America (Fig. 6). While additional theoretical analysis is required to understand whether this vertical wave coupling over North America is due to planetary wave reflection^3,26,31^ or other mechanisms, these results provide evidence that the vertical coupling between stratospheric and tropospheric waves plays a key role in the North American cold anomalies following strong stratospheric wave events.

Finally, since the lowest SAT over North America on February 13, 2021 is followed by a local minimum of the stratospheric wave index on February 18 (Fig. 3e), we use the nudging simulations to test the causal relationship between surface cold wave events and concurrent weak stratospheric wave activity. Weak stratospheric wave events in the NUDG simulations show similar results as strong wave events but with opposite signs (Supplementary Fig. 6). The cold anomalies before and around weak wave events largely diminish in the NUDG simulation, implying a tropospheric rather than stratospheric origin for the North American cold. This suggests a predominant role of tropospheric variability in the February 2021 cold spell, consistent with previous studies^5,9^.

## Discussion

Considering the proposed linkage between stratospheric polar vortex stretching and cold extremes over North America such as the February 2021 cold wave^3^, we have investigated the robustness and mechanisms of the contributions of extreme stratospheric wave events to surface cold anomalies. Using observations and the historical simulations of 30 CMIP6 models, we show that strong stratospheric wave events, due to the constructive interference between transient and climatological planetary waves (Supplementary Fig. 1), are characterized by a polar vortex displacement to Eurasia, followed by an increased risk of cold air outbreaks over North America 5–25 days later (Fig. 4). This suggests that strong stratospheric wave events can be used as a predictor for North American cold events on sub-seasonal timescales.

Importantly, extreme stratospheric wave events are accompanied by intraseasonal fluctuations between warm and cold spells over North America (Fig. 3), which are distinct from the well-known surface influences of the stratosphere such as SSWs and strong vortex events that increase the intraseasonal persistence of weather regimes^12–18,43^ or the lower-stratospheric planetary wave reflection events^3,26^. While SSWs are followed by negative AO on weekly to monthly timescales^12,14,43^, the cold anomalies over North America and positive NAO following strong wave events largely vanish 25 days later (Fig. 3). This relatively short timescale of strong wave events implies a distinct physical mechanism from SSWs^29^. If one takes a time average of 20 days or longer around the strong wave event onset, the North American warming precursor would overwhelm the following cooling signal, leading to weak surface warming associated with strong wave events. This is partly why this lead-lag relationship between strong stratospheric wave activity and surface cooling received little attention in the literature^28,44^. Moreover, under certain circumstances, a weak stratospheric wave event may transition to a strong wave event that in turn precedes surface cold anomalies, which may be thought of as an intraseasonal mode of stratosphere-troposphere oscillation^30^. Although some strong stratospheric wave events are indeed preceded by negative planetary wave indices, the stratospheric conditions prior to strong wave events exhibit a very large uncertainty, indicating a small signal-to-noise ratio beyond the timescale of a strong wave event (Supplementary Fig. 7).

We further demonstrate in observations and idealized nudging simulations that the vertical coupling between stratospheric and tropospheric waves is key to the observed North American cooling following strong stratospheric wave events. Strong wave events feature upward and eastward wave activity fluxes in the lower stratosphere over Siberia and downward wave activity fluxes over northern North America, and the latter corresponds to an anomalous tropospheric trough (Fig. 5b, c) and associated cold anomalies over North America (Fig. 6) 5–25 days later. The upward fluxes over Siberia might also be traced back to tropospheric precursors that further extend the timescale of predictability. Future work may use the Subseasonal to Seasonal Prediction (S2S) data set^19,35^ or linear inverse model (LIM)^5^ to quantify the contributions of extreme stratospheric wave events to surface variability as compared with other sources of intraseasonal predictability. These findings can potentially improve the predictability of severe winter cold events in the U.S. and Canada and consequently benefit the transportation sector^45,46^, energy planning and use^47,48^, and human health^49,50^.

## Methods

### Reanalysis data and CMIP6 models

We use atmospheric and surface data from the fifth generation of atmospheric reanalysis from the European Centre for Medium-Range Weather Forecasts (ERA5)^51^. The daily data at a resolution of 1.5^∘^ × 1.5^∘^ is analyzed for the extended boreal winter from November to March over the period 1950–2021. After detrending, we remove the seasonal cycle in the data, which is defined as the time mean and first two harmonics of the full-year climatology.

We also employ historical simulations of 30 CMIP6 models. The daily data are examined for the period of 1950–2014 (except for GISS-E2-2-G in 1970–2014 due to limited data availability). All model data are bilinearly interpolated to a common 1.5^∘^ × 1. 5^∘^ grid. Only a single member of each model ensemble is analyzed here. See the list of CMIP6 models, the ensemble members used, and vertical resolution in Supplementary Table 1.

### Definition and statistics of extreme stratospheric wave events

We use a planetary wave index that is simple and readily applicable to reanalyses and climate model outputs. Extreme stratospheric wave events are defined based on empirical orthogonal function (EOF) analysis of the geopotential height at 10 hPa, for ERA5 reanalysis and each CMIP6 model individually (Supplementary Fig. 1). The planetary wave index is obtained as the standardized principal component of the first EOF of the zonally asymmetric 10 hPa geopotential height north of 20^∘^N, weighted by the square root of the cosine of latitude.

For both the reanalysis and models, a weak stratospheric wave event is detected as the consecutive days when the planetary wave index is below the 5th percentile, and a strong wave event corresponds to the consecutive days when the index is above the 95th percentile. No minimum duration is required for an event. The first day satisfying the threshold criterion is referred to as day 0 of the event, day -5 denotes 5 days before day 0, and day 5 denotes 5 days after day 0. This results in 89 weak wave events and 93 strong events out of 71 winters in ERA5, at the frequency of 1.25 weak and 1.31 strong events per year. The CMIP6 model ensemble produces an average frequency of 1.29 ± 0.16 weak and 1.32 ± 0.21 strong events per year. The uncertainty is estimated by the SD across the CMIP6 models. Moreover, in the SC-WACCM4 simulations described below, 56 weak and 64 strong wave events are detected out of 49 winters, at the frequency of 1.14 weak and 1.31 strong events per year. This indicates that the frequency of extreme stratospheric wave events is consistent among reanalysis and models, and hence the models represent a valid tool for studying these events.

### SC-WACCM4 and nudging experiments

SC-WACCM4 is the stratosphere-resolving atmospheric component of the National Center for Atmospheric Research (NCAR) Community Earth System Model version 1.2 (CESM1), with specified chemistry to reduce the computational cost without changing the climatology and variability of the atmospheric circulation in the troposphere and stratosphere^39^. SC-WACCM4 has 66 vertical levels and a horizontal resolution of 1.9^∘^ × 2.5^∘^, with a model lid at 5.1 × 10^−6^ hPa.

Two SC-WACCM4 experiments, a control run (CTL) and a nudged run (NUDG), are employed to examine the surface signatures of extreme stratospheric wave activity. The two experiments are the same as those from a previous study^52^. In CTL, the boundary condition of the model is prescribed by the repeating climatological seasonal SST and SIC, which are obtained from the CESM1-WACCM4 historical outputs from the CMIP5 and averaged during 1980–1999 from 7 ensemble members. The nudged run is the same as CTL, but a nudging method was applied. Specifically, the temperature, zonal wind, and meridional wind above 90 hPa were nudged toward those in the CTL with a damping time scale of 6 h. The fields were fully nudged above 54 hPa, with no nudging applied below 90 hPa and a linearly tapering region in between. The nudging was performed at every time step of the model integration, but the target states from the CTL run were read in every 6 h, and the model fields were nudged toward the linear interpolation between consecutive target states, which, in this case, were the time-evolving CTL simulation.

By the experimental design, the evolution of the stratospheric circulation is largely the same (but not identical) in NUDG and CTL. Although the surface boundary condition in NUDG is the same for the troposphere as in CTL, the small differences in the stratosphere resulting from nudging produce distinct trajectories in tropospheric weather from CTL due to the chaotic nature of weather systems. Thus, the tropospheric circulation in NUDG can be regarded as a distinct realization of weather systems, including the downward influence from the stratospheric variability in the CTL run. We note that the nudging technique does not change the winter climatology or standard deviation of SAT over North America. More details of the experimental design and the evaluation of nudging method can be found in a previous study^53^. Note that instead of nudging only the zonal mean fields^53^, the full fields were nudged^52^ in the current study such that the same stratospheric events are found in both CTL and NUDG experiments. The model experiments were integrated for 60 years for both CTL and NUDG, and the last 50 years are analyzed.

### Plumb wave activity flux

We use the 3D Plumb wave activity flux to describe zonal, meridional, and vertical wave propagation of quasi-stationary waves^54^.

1 $$\{{F}^{\lambda },{F}^{\phi },{F}^{z}\}= 	\, p\cos (\phi )\left\{{v{}^{{\prime} }}^{2}-\frac{1}{fa\cos (\phi )}\frac{\partial ({v}^{{\prime} }{\Phi }^{{\prime} })}{\partial \lambda },\right.\\ 	\left.-{u}^{{\prime} }{v}^{{\prime} }+\frac{1}{fa\cos (\phi )}\frac{\partial ({u}^{{\prime} }{\Phi }^{{\prime} })}{\partial \lambda },\frac{f}{\partial \tilde{T}/\partial z+\kappa \tilde{T}/H}\left[{v}^{{\prime} }{T}^{{\prime} }-\frac{1}{fa\cos (\phi )}\frac{\partial ({T}^{{\prime} }{\Phi }^{{\prime} })}{\partial \lambda }\right]\right\}$$

where *λ* is longitude, *ϕ* is latitude, *z* is height, and *p* is pressure. *u* is the zonal wind, *v* is the meridional wind, *T* is temperature, and Φ is geopotential height. *f* is the Coriolis parameter. *a* is Earth’s radius. *κ* is the specific gas constant of dry air divided by the specific heat of dry air. $\tilde{T}$ denotes the domain average of temperature. *H* is the log-pressure scale height. Primes denote the deviations from zonal means.

The vertical component of the Plumb flux approximately corresponds to the vertical phase tilt of a planetary wave: the upward Plumb flux corresponds to the typical westward phase tilt of a trough or ridge with increasing height, and the downward Plumb flux coincides with an eastward phase tilt with height.

### Supplementary information

Supplementary Information

## Data Availability

The ERA5 reanalysis is available at https://www.ecmwf.int/en/forecasts/datasets/reanalysis-datasets/era5. The CMIP6 outputs used in this study can be obtained from the CMIP archive at https://esgf-node.llnl.gov/projects/esgf-llnl. The SC-WACCM4 data used in this study is available via figshare at 10.6084/m9.figshare.22682344.v1.

## Code availability

All plots and analyses were carried out using NCAR Command Language (NCL) version 6.3.0 and 6.6.2. All code files are available upon request to the corresponding authors.
