# Supplementary material for: Extreme stratospheric wave activity as harbingers of cold events over North America
Source: Commun Earth Environ. 2023 May 27;4(1):187. doi: 10.1038/s43247-023-00845-y (PMC11041758; doi:10.1038/s43247-023-00845-y)
Supplement: Supplementary file 1 — Supplementary Information [file 43247_2023_845_MOESM1_ESM.pdf]

Supplementary Information for "Extreme Stratospheric Wave Activity as Harbingers of Cold Events over North America"

Xiuyuan Ding[1*], Gang Chen[1*], Pengfei Zhang[2], Daniela I.V. Domeisen[3,4] and Clara Orbe[5]

[1*]Department of Atmospheric and Oceanic Sciences, University of California, Los Angeles, Los Angeles, CA, USA.
[2]Department of Meteorology and Atmospheric Science, The Pennsylvania State University, University Park, PA, USA.
[3]University of Lausanne, Lausanne, Switzerland.
[4]Institute for Atmospheric and Climate Science, ETH Zurich, Zurich, Switzerland.
[5]NASA Goddard Institute for Space Studies, New York, NY, USA.

*Corresponding author(s). E-mail(s): dingxy@ucla.edu; gchenpu@ucla.edu;

Contents of this file

The Supplementary Information (SI) includes

1. Tables S1
2. SI Figures: Supplementary Figs. 1 to 8.

SI Table and Figures

Supplementary Table 1 List of the CMIP6 models used in this study

Model	No. of Vertical Levels	Lid Height (hPa)	Ensemble Member
ACCESS-CM2	85	0.0054	r1i1p1f1
AWI-ESM-1-1-LR	47	0.011	r1i1p1f1
BCC-ESM1	26	2.19	r1i1p1f1
CESM2	32	2.25	r1i1p1f1
CESM2-WACCM	70	4.5e-6	r1i1p1f1
CNRM-CM6-1	91	0.014	r1i1p1f2
CNRM-CM6-1-HR	91	0.014	r1i1p1f2
CNRM-ESM2-1	91	0.014	r1i1p1f2
CanESM5	49	1	r1i1p1f1
EC-Earth3	91	0.01	r1i1p1f1
FGOALS-f3-L	32	2.16	r1i1p1f1
FGOALS-g3	26	2.19	r1i1p1f1
GFDL-ESM4	49	1	r1i1p1f1
GISS-E2-1-G	40	1	r1i1p1f2
GISS-E2-2-G	102	0.002	r1i1p1f1
HadGEM3-GC31-LL	85	0.0054	r1i1p1f3
HadGEM3-GC31-MM	85	0.0054	r1i1p1f3
INM-CM4-8	21	10	r1i1p1f1
INM-CM5-0	73	0.2	r1i1p1f1
IPSL-CM6A-LR	79	0.011	r1i1p1f1
KACE-1-0-G	85	0.0054	r1i1p1f1
MIROC-ES2L	40	3	r1i1p1f2
MIROC6	81	0.004	r1i1p1f1
MPI-ESM-1-2-HAM	47	0.01	r1i1p1f1
MPI-ESM1-2-HR	95	0.01	r1i1p1f1
MPI-ESM1-2-LR	47	0.01	r1i1p1f1
MRI-ESM2-0	80	0.01	r1i1p1f1
NorESM2-LM	32	3	r1i1p1f1
NorESM2-MM	32	3	r1i1p1f1
UKESM1-0-LL	85	0.0054	r1i1p1f2

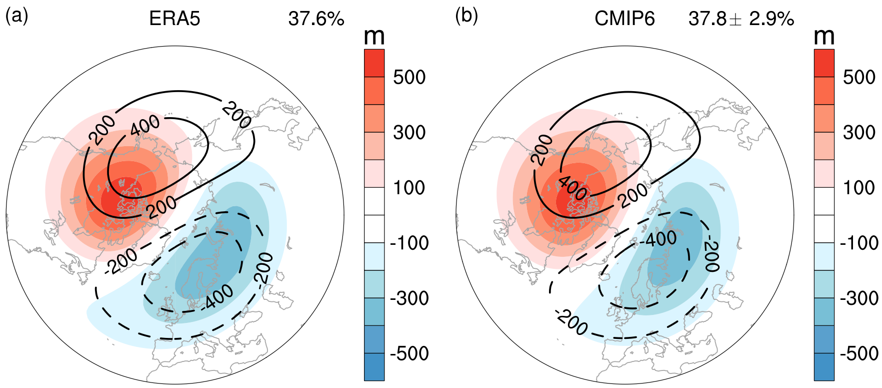

Supplementary Fig. 1 Leading EOFs of the zonally asymmetric component of 10-hPa geopotential height. a-b, The leading EOF pattern for ERA5 (**a**) and the multi-model mean of leading EOFs of CMIP6 models (**b**). The climatological wave pattern is shown in black contours. The percentage of variance explained by the leading EOF is depicted at the upper right corner. The uncertainty of CMIP6 models is given as the SD among 30 models.

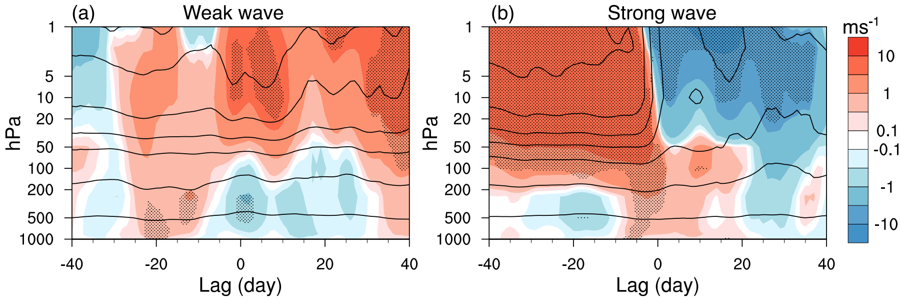

Supplementary Fig. 2 Zonally averaged high-latitude zonal wind for extreme stratospheric wave events in ERA5. a-b, Zonal wind (contours at 10 m s^{-1}, anomalies shaded) averaged over 60°–90°N during weak stratospheric wave events (**a**) and strong stratospheric wave events (**b**). A 5-day running average is applied. Stippling indicates the regions where the anomalies are significant at the 95% confidence level based on the Student's t-test.

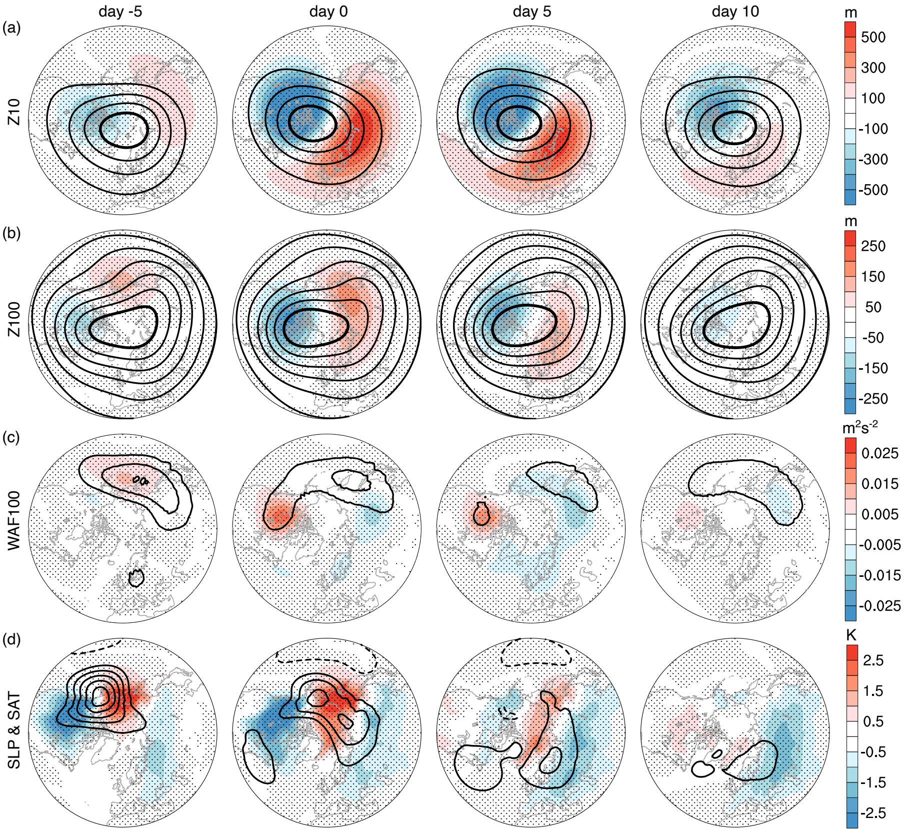

Supplementary Fig. 3 Weak stratospheric wave events in CMIP6 models. As in Fig. 1, but for the composites in CMIP6 models. See details of CMIP6 models in Methods.

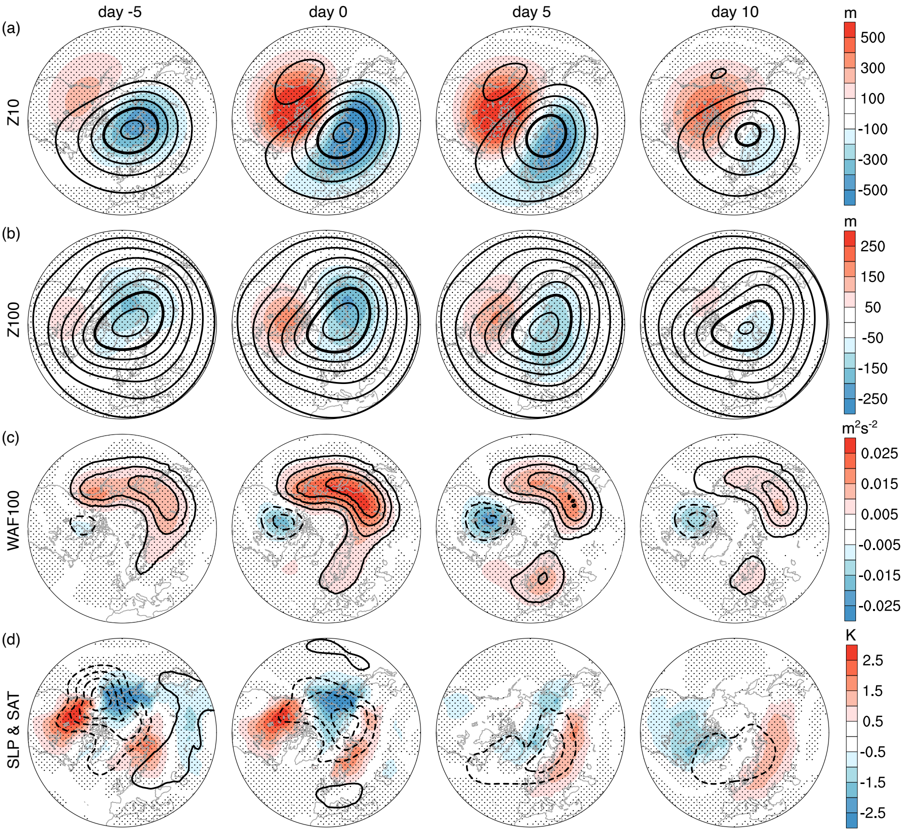

Supplementary Fig. 4 Strong stratospheric wave events in CMIP6 models. As in Fig. 2, but for the composites in CMIP6 models. See details of CMIP6 models in Methods.

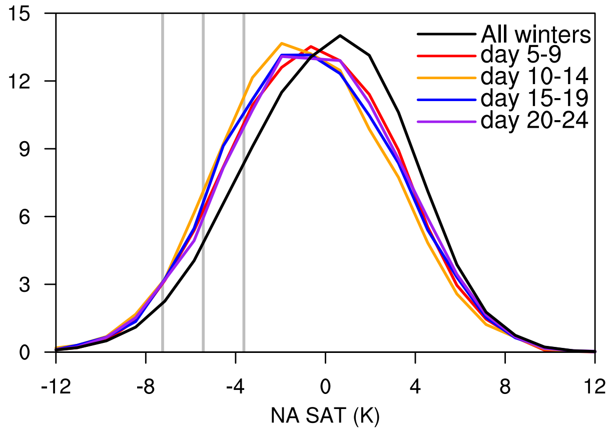

Supplementary Fig. 5 PDF of NA SAT anomalies following strong stratospheric wave events in CMIP6. As in Fig. 4d, but for finer time windows (i.e., day 5–9, day 10–14, day 15–19, and day 20–24). The vertical gray lines denote -1, -1.5, and -2 SD of NA SAT anomalies in all the winter days.

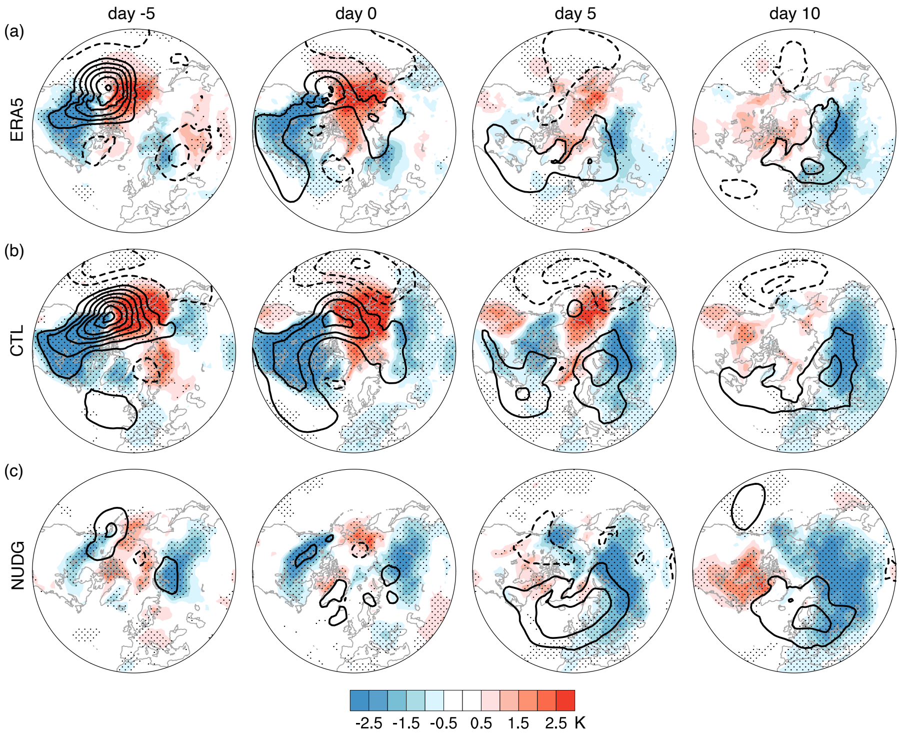

Supplementary Fig. 6 Surface signatures of weak stratospheric wave events in SC-WACCM4. Composites of anomalous SLP (contours at 2 hPa intervals) and SAT (shading) for the CTL (**b**) and NUDG (**c**) experiments of SC-WACCM4, as compared with ERA5 (**a**, same as Fig. 1d). Stippling indicates the regions where the SAT anomalies are significant at the 95% confidence level based on the Student's *t*-test.

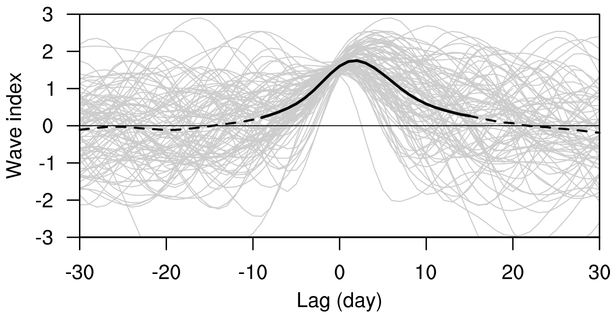

Supplementary Fig. 7 Evolution of the planetary wave index linked to strong stratospheric wave events in ERA5. Composites of all the events are depicted as the black line and individual events are shown in light gray. Solid parts of the line represent the composites significant at the 95% confidence level based on the Student's *t*-test.

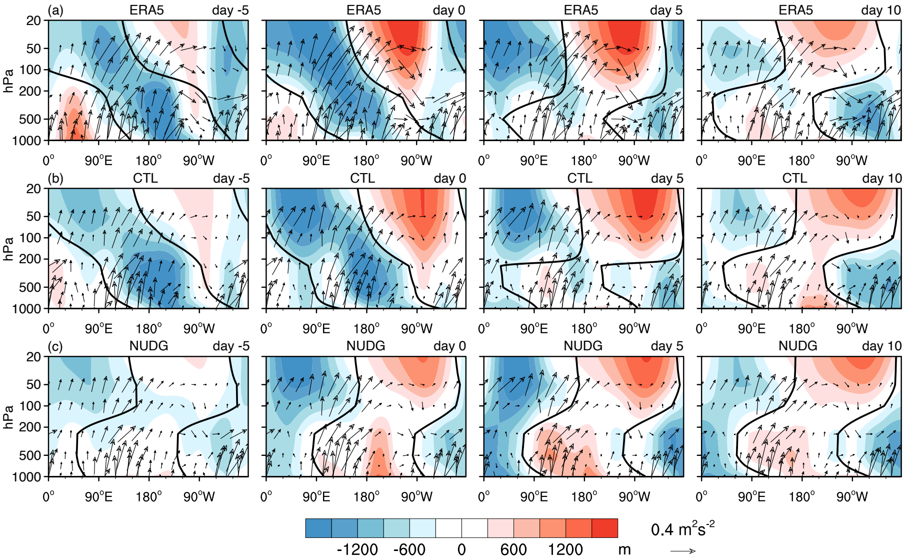

Supplementary Fig. 8 Vertical wave coupling during strong stratospheric wave events. As in Fig. 5, but for composites of the total field of anomalous geopotential height (zonal mean plus zonal asymmetry, shading) and the vertical and zonal components of absolute Plumb wave activity flux (climatology plus anomalies, vector).
